# Sources of ambient PM_2.5_ exposure in 96 global cities

**DOI:** 10.1016/j.atmosenv.2022.119234

**Published:** 2022-10-01

**Authors:** Mei W. Tessum, Susan C. Anenberg, Zoe A. Chafe, Daven K. Henze, Gary Kleiman, Iyad Kheirbek, Julian D. Marshall, Christopher W. Tessum

**Affiliations:** a Department of Agricultural and Biological Engineering, University of Illinois at Urbana-Champaign, Urbana, IL, United States; b Department of Environmental and Occupational Health, George Washington University, Washington, DC, United States; c C40 Cities Climate Leadership Group Inc., New York, NY, United States; d Department of Mechanical Engineering, University of Colorado, Boulder, CO, United States; e Orbis Air, LLC, Concord, MA, United States; f Department of Civil and Environmental Engineering, University of Washington, Seattle, WA, United States; g Department of Civil and Environmental Engineering, University of Illinois at Urbana-Champaign, Urbana, IL, United States

**Keywords:** Environmental policy, Fine particulate matter, Air quality, Pollution, Metropolitan, Air quality modeling, Chemical transport modeling

## Abstract

To improve air quality, knowledge of the sources and locations of air pollutant emissions is critical. However, for many global cities, no previous estimates exist of how much exposure to fine particulate matter (PM_2.5_), the largest environmental cause of mortality, is caused by emissions within the city vs. outside its boundaries. We use the Intervention Model for Air Pollution (InMAP) global-through-urban reduced complexity air quality model with a high-resolution, global inventory of pollutant emissions to quantify the contribution of emissions by source type and location for 96 global cities. Among these cities, we find that the fraction of PM_2.5_ exposure caused by within-city emissions varies widely (μ = 37%; σ = 22%) and is not well-explained by surrounding population density. The list of most-important sources also varies by city. Compared to a more mechanistically detailed model, InMAP predicts urban measured concentrations with lower bias and error but also lower correlation. Predictive accuracy in urban areas is not particularly high with either model, suggesting an opportunity for improving global urban air emission inventories. We expect the results herein can be useful as a screening tool for policy options and, in the absence of available resources for further analysis, to inform policy action to improve public health.

## Highlights

• The contributions of different air pollution sources vary widely among cities.
• The contributions of within-city vs. external emission sources to a city's air pollution also varies widely among cities.
• The contributions above cannot be accurately predicted without air quality modeling.
• An opportunity exists to improve global emission inventories in urban areas.

## 1 Introduction

Air pollution is the greatest single environmental health risk worldwide. According to the World Health Organization, ambient air pollution prematurely kills 7 million people per year (World Health Organization, 2021), with an estimated economic cost of ∼ $3 trillion USD, or 3.3% of global GDP (Myllyvirta, 2020). Among air pollutants, fine particulate matter (PM_2.5_) has the largest health impact in monetized terms—more than half of the global population is exposed to annual-average ambient concentrations exceeding the first interim target from the World Health Organization, 35 μg/m^3^ (Health Effects Institute, 2019). Efforts to reduce PM_2.5_ concentrations have not been uniformly successful (UNEP, 2021).

Making effective plans for improving air pollution requires prioritization, which in turn requires knowing the emission sources that contribute most to poor ambient air quality and to the resulting health effects. However, the complexity of the atmospheric system and of the human and natural systems that cause emissions make that task challenging; in many cases it can be difficult for scientists and policymakers to determine which sources to target to maximally reduce the population exposure.

Air quality models and other decision support tools that relate pollution emissions to the resulting ambient concentrations and health impacts can be important for designing effective policies to improve air quality. Mechanistically detailed Eulerian chemical transportation models (CTMs) are considered well-suited for this purpose (Thunis et al., 2019), but owing to high requirements for user training, computational resources, and input data, are often unavailable for urban-level policy analysis. Multiple reduced-complexity air quality models (RCMs) have been designed to fill this gap (Levy et al., 2009; Heo et al., 2017; Casey et al., 2018; Mikati et al., 2018). Among these RCMs, the Intervention Model for Air Pollution (InMAP; Tessum et al., 2017) has proven useful in health impact assessment and environmental justice applications (Goodkind et al., 2019; Tessum et al. 2019, 2021; Tschofen et al., 2019; Hill et al., 2019; Thind et al., 2019; Liu et al., 2019) owing in part to its use of a variable spatial resolution computational grid which focuses computational effort on areas with high population density. The recent creation of a global-through-urban version of the InMAP model (Thakrar et al., 2022) now provides the opportunity to estimate the exposure consequences of primary and secondary PM_2.5_ concentrations at high spatial resolution in nearly all densely populated areas globally.

Metropolitan-level policy can play an important role in urban air quality (Friedman et al., 2021; Streets et al. 2007; Tonne et al. 2008; Slovic and Ribeiro, 2018; Izquierdo et al. 2020), but policymakers in many global cities have not previously had detailed information regarding where their ambient PM_2.5_ pollution comes from. Common initial questions from policy makers, in considering how to address air pollution, include: (1) Source apportionment: which sources (electricity generation, transportation, industry, etc.) contribute substantially to ambient pollution levels? and (2) Local influence and authority: how much pollution is generated within the city versus transported in from outside? Previous globally-scoped studies have provided this information at national or subcontinental resolution (e.g., Lelieveld et al. 2015; Anenberg et al., 2019)---which is of limited use for decision-making at the urban level---or have provided information on the sources contributing to pollution in cities based on a relatively low-resolution emissions inventory but no information regarding whether those sources are located within or external to the city boundaries (McDuffie et al. 2021).

Here, we use InMAP to provide scoping-level answers to the two questions above for 96 global cities. Specifically, we estimate the contribution of 12 emission source sectors, both within and outside of the city boundaries, to concentrations of primary (i.e., directly emitted) and secondary (i.e., formed in the atmosphere from primary emissions of gaseous pollutants) PM_2.5_. We also evaluate model performance in these urban areas and discuss opportunities for future improvements in model accuracy. For many of the cities we study, information provided herein is the only quantitative information that exists regarding the within-city vs. out-of-city contribution to ambient PM_2.5_. Results reported here provide both information for stakeholders and an analysis of opportunities to improve the accuracy of results in future work.

## 2 Methods

Fig. S1 provides an overview of the input data, output data, and modeling tools used in this study, which are described in detail below.

### 2.1 PM_2.5_ emission source estimation inventory data

We study 96 cities that are members of C40, a network of mayors of global cities dedicated to delivering action on climate change (C40, 2021). For each city, we consider anthropogenic, biogenic, mineral dust, soil denitrification, and biomass burning emissions. For anthropogenic emissions, we use data from the Community Emissions Data System (CEDS) for year 2014 with eight sectors (Table 1): non-combustion agriculture (AGR); energy transformation and extraction (ENE); industrial combustion and processes (IND); surface transportation (road, rail, other) (TRA); residential, commercial, and other (RCO); solvents (SLV); waste disposal and handling (WST); and international shipping (SHP) (Hoesly et al., 2018). The CEDS emission species used here and their mappings to InMAP species are summarized in Table S1. The CEDS emissions dataset is available at 0.5 × 0.5° spatial resolution, meaning that a single emissions grid cell is larger than many of the cities we study. Section 2.2 describes our methods for downscaling these emissions data to produce higher-resolution estimates.

**Table 1:** Sectors of anthropogenic emissions from the Community Emissions Data System (Hoesly et al., 2018) and concordance with spatial surrogates for downscaling.

Sector	Specification	Spatial Surrogate
Non-combustion agricultural sector (AGR)	manure management, soil emissions, rice cultivation, enteric fermentation, and other	Agricultural sector
Energy transformation and extraction (ENE)	electricity production, heat production, other energy transformation, related fugitive emissions, and fossil fuel fires	Energy generation
Industrial combustion and processes (IND)	combustion for manufacturing of goods and minerals and for construction, production of cement, lime, and “other minerals”, mining, chemical production, paint application, wood, pulp, and paper products	Industrial sector
Surface transportation (road, rail, other) (TRA)	air, road, rail, and water transportation	Roadways
Residential, commercial, and other (RCO)	commercial-institutional, residential, agriculture-forestry-fishing, and other-unspecified emissions	Population
Solvents (SLV)	used in degreasing and cleaning	Industrial sector
Waste disposal and handling (WST)	solid waste disposal, waste combustion, wastewater handling, and other	Population
International shipping (SHP)	VOCs from oil tanker loading/leakage	Waterways

We use biogenic volatile organic chemical (VOC) emissions generated by the MEGAN model (Guenther et al., 2006), including the individual VOC components that are considered secondary organic aerosol (SOA) precursors by GEOS-Chem. We use mineral dust emissions generated by the “DustDead” GEOS-Chem algorithm (Zender et al., 2003). The DustDead model simulates emissions of mineral dust that are mobilized by wind (excluding road dust). The “EMIS_DST1” and “EMIS_DST2” variables were used in this study to represent primary PM_2.5_ emissions. We use soil NO_x_ emissions from the GEOS-Chem soil NO_x_ extension (Hudman et al., 2012). These emissions are at 0.25 × 0.3125° spatial resolution and for year 2016, downloaded from the GEOS-Chem FTP website (GEOS-Chem authors, 2019).

We use biomass burning emissions from the fourth generation Global Fire Emissions Database (GFED4; Giglio et al., 2013) at 0.25 × 0.25° spatial resolution for year 2016. These emissions are separate from domestic biomass burning for residential energy use, which is included in the CEDS dataset.

### 2.2 Spatial surrogates

We downscale anthropogenic emissions from the native 0.5 × 0.5° CEDS spatial resolution to InMAP grid cells—which vary in size with a minimum edge length of 0.039 × 0.03125°—using surrogate spatial data, which allows us to represent the spatial distribution of emissions within each CEDS grid cell. We do not apply additional spatial processing to non-anthropogenic emissions. To spatially downscale anthropogenic emissions, we employ spatial datasets that are global in scope and freely available, allowing us to scale our approach to a large number of cities. In cases where the datasets provide no information for a given surrogate within a given city—for example, some cities do not have agricultural areas within their boundaries—we assume emissions are evenly distributed throughout the city area. We also assume all emission sources except electricity generation occur at ground-level. Spatial surrogates used for each CEDS emission sector are as follows:

For *energy transformation and extraction*, we use a database of SO_2_ emissions from global electricity generating units (EGUs) (Tong et al., 2018). We use the spatial distribution of SO_2_ emissions (rather than another pollutant) because SO_2_ emissions are responsible for the vast majority of overall health impacts from EGUs (Fann et al., 2012). EGUs typically have tall emissions stacks, and their emissions plumes often continue to rise after release, owing to their upward velocity exiting the stack and their higher temperature relative to surrounding air. To incorporate stack height and plume rise, we assume that EGUs have stack parameters equal to mean values for EGUs in the year-2014 US EPA National Emissions Inventory (US EPA, 2016) as processed by Tessum et al. (2019): 63.5 m stack height, 4.1 m stack diameter, 519.2 K emission temperature and 24.7 m/s emission velocity. (There is no global database of EGU stack properties.)

For *surface transportation*, we create a spatial surrogate using a weighted average of roadway lengths of OpenStreetMap (OSM) roadways in each CEDS grid cell. We use the following weighted average of OSM roadway types: 36% motorways, 21% trunk roads, 18% primary roads, 9% secondary roads, 1% tertiary, unclassified, and service roads, and 14% residential roads. This weighting is derived from U.S. data on urban road uses (US DOT FHA, 2017); the taxonomy of roadway types is described in Table S2 (OpenStreetMap contributors, 2019).

For *international shipping****,*** we create a spatial surrogate from the combined length of OSM features tagged as river, riverbank, pier, ferry, ferry terminal, boat, and mooring. For *non-combustion agriculture*, we create a spatial surrogate from the combined length of OSM features tagged as farm, farmland, or vineyard (OpenStreetMap contributors, 2017). For *industrial combustion and processes* and *solvents*, we create a spatial surrogate from the combined area of OSM features tagged as industrial buildings or “industrial” or “quarry” land use. For the remaining categories (*residential, commercial, and other*, and *waste disposal and handling*), we assume a spatial distribution similar to population density, which we represent using the year-2020 projected population from the WorldPop database (TatemWorldPop, 2017).

### 2.3 InMAP air quality modeling

**Air quality model description:** The Intervention Model for Air Pollution (InMAP) is a mechanistic reduced-complexity air quality model (RCM) that estimates annual-average changes in primary and secondary PM_2.5_ concentrations attributable to annual changes in emissions of PM_2.5_ and its precursors. InMAP leverages pre-processed physical and chemical information from the output of a comprehensive CTM (in this case, GEOS-Chem) and uses a variable spatial-resolution computational grid to perform simulations that are several orders of magnitude less computationally intensive than conventional CTMs, yet with spatial resolution that is higher than is typically possible using a conventional CTM for a given domain. Conventional CTMs create a three-dimensional Eulerian grid and simulate changes in pollutant concentration in each cell at a high temporal resolution (<1 h) based on physical transport via wind flow and plume rise, emissions, physical removal (e.g., deposition), and interdependent non-linear physico-chemical transformation pathways. In contrast, InMAP uses time-averaged transport and reaction rates in its algorithms for emission, plume rise, transport, transformation, and removal of atmospheric pollution. To reduce computational intensity, the algorithms are in some cases simplified relative to similar algorithms in a conventional CTM; these simplified representations are calibrated using output from a conventional CTM (GEOS-Chem).

In addition to the emissions and population data described in Section 2.1, InMAP requires information on meteorological characteristics and on chemical transport and reaction rates. We use meteorological and background chemistry inputs generated from the outputs of the GEOS-Chem global atmospheric chemical transport model (CTM) simulation for the year 2016, with a base spatial resolution of 2 × 2.5° and regional nests over Asia, Europe, and North America at 0.5 × 0.625° spatial resolution. The GEOS-Chem simulation uses the SOA_SVPOA chemical mechanism with standard emissions inputs as processed by the HEMCO emissions processor. Further details regarding the GEOS-Chem configuration are described by Thakrar et al. (2021).

This is the first detailed application of InMAP to global cities, but it has previously been used to study PM_2.5_ air pollution in the US, including racial-ethnic disparities in exposure (Tessum et al., 2019, 2021), and exposure to air pollution from agriculture (Hill et al., 2019), electricity (Thind et al., 2019), and freight (Liu et al., 2019). Further details regarding the InMAP model, including model formulation and performance evaluation, for the US and globally, are described in detail elsewhere (Tessum et al., 2017; Thakrar et al., 2022).

**Air quality model application:** First, we estimate, for each city, the annual average total PM_2.5_ concentrations and the contributions from each of the 12 source sectors. Here, our spatial domain is global, so we refer to these as the “global” simulations. We configure InMAP (version 1.9.6) to use a variable-resolution grid with 2 × 2.5° resolution for the largest cells, each of which are allowed to split into 4 smaller cells up to 6 times recursively, for a minimum grid cell size of 0.031 × 0.039° (about 3 × 4 km^2^ at the equator). The resulting grid (Fig. S2) was created by recursively splitting any grid cell containing more than 100,000 people or containing more than 55 million people per square degree in any part of the cell. These 12 simulations (one per source sector), each require ∼20 h on a circa-2022 computer with 16 CPU cores.

Next, we estimate, for each city, the same two parameters as in the first step (total PM_2.5_ and contributions from each sector) but in this case only considering within-city emissions (i.e. emissions originating within a city boundary provided by city officials for each of the 96 cities). For the resulting 1152 city simulations, we use the same InMAP configuration as above.

### 2.4 Data analysis

For each source sector, we estimate exposure impacts from emissions originating outside of each city by subtracting population-weighted concentrations caused by emissions within the city from total population-weighted concentrations. In this manner we obtain the fraction of total PM_2.5_ concentrations caused by within-city emissions as well as the fraction of total PM_2.5_ concentrations caused by different emission sources located either within or outside of the city.

Because the air quality model simulations we perform here require a substantial amount of expertise, time and computational resources, we investigate whether patterns exist in the underlying results that could potentially allow extrapolation beyond the 96 cities we studied. To do so, we analyze the relationship between the fraction of PM_2.5_ originating from within city sources and various city characteristics such as city population, gross domestic product (GDP), city area, and “population buffer fraction”. We define population buffer fraction as the city population divided by the total population within a radius of 200-km from the city centroid. The goal of this supplementary analysis is to explore whether there might exist a straightforward method to reproduce the results shown here without extensive air quality modeling.

### 2.5 Model evaluation

We evaluate InMAP model prediction accuracy of total PM_2.5_ concentration by comparing InMAP population-weighted predictions for cities against measurements from the WHO ambient (outdoor) air pollution database (World Health Organization, 2016), which provides PM_2.5_ measurements for 53 of the 96 cities studied here. (The WHO data we use provides the average of all monitors in a city rather than values from individual monitors and combines measurements collected in different years.) We assess InMAP model performance using metrics including mean bias (MB), mean error (ME), mean fractional bias (MFB), mean fractional error (MFE), and coefficient of determination (r^2^). Definitions of these metrics are in Table S3. We also use the same metrics to evaluate the global GEOS-Chem model predictions as configured and run by Thakrar et al. (2021) against the same PM_2.5_ measurements in the same cities. In addition, we compare InMAP model predictions of total PM_2.5_ concentrations against satellite-based predictions of PM_2.5_ concentrations in 91 global cities (Southerland et al., 2021), and predictions by McDuffie et al. (2021) in 43 global cities.

Although there do not exist city-specific estimates of PM_2.5_ source apportionment for all of the cities studied here, we compare our estimates for fractional contributions of six similar emission sources with those reported by McDuffie et al. (2021) among 43 global cities. We also compare our estimates for fractions of total PM_2.5_ and fractions caused by eight emission sources generated by within-city emissions with those reported by Thunis et al. (2021) among 17 European cities estimated by the European Commission.

## 3 Results

### 3.1 InMAP PM_2.5_ concentration and source analysis

InMAP model results provide estimates (population-weighted concentrations) for each city of primary and secondary PM_2.5_, chemically-speciated by source type for within-city versus outside-city emissions. Results are summarized next, with full results for each city in supporting dataset S1.

The median (interquartile range [IQR]) predicted concentration among the 96 cities is 17 (8–40) μg/m^3^, of which we estimate 44% (25%–62%) is primary (the rest is secondary), and 33% (21%–52%) comes from within-city emissions (the rest comes from sources outside the urban boundary; Table S4).

**Fig. 1:**
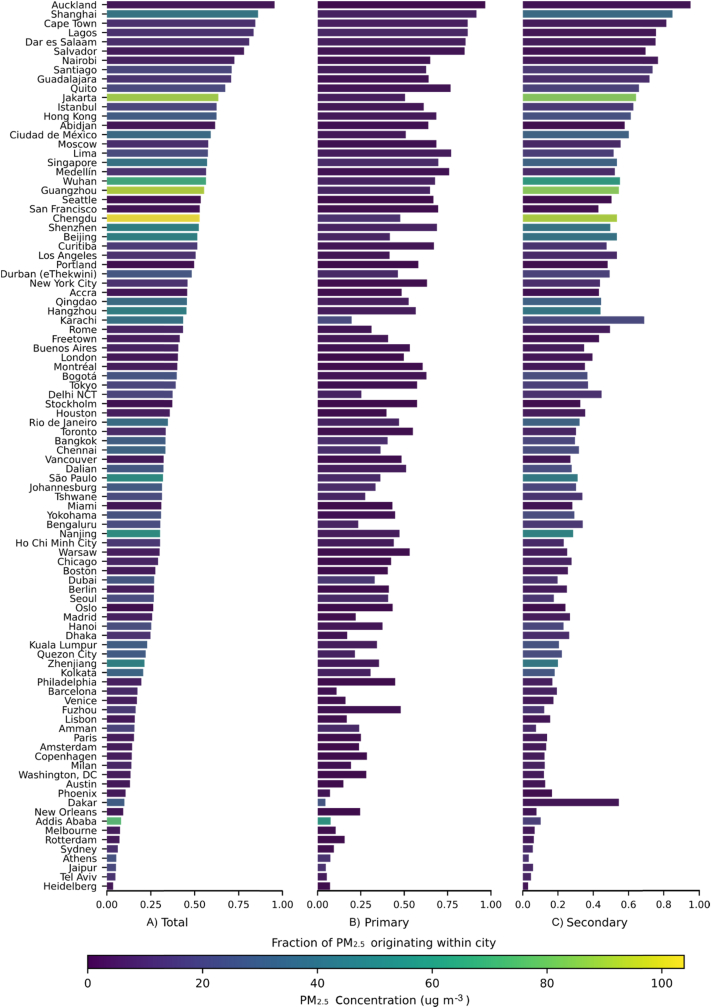
Fractions of PM_2.5_ originating from within city sources for A) total PM_2.5_, B) primary PM_2.5_ and C) secondary PM_2.5_ among 96 global cities. Color scales represent population-weighted PM_2.5_ concentration (μg/m^3^). (For interpretation of the references to colour in this figure legend, the reader is referred to the Web version of this article.)

Concentration estimates for each city (Fig. 1) demonstrate substantial variability among urban areas, in terms of concentrations as well as the proportion that is primary vs secondary particulate matter. The highest levels of total PM_2.5_ as predicted here are mainly in Asian cities. As described in Section 3.3, InMAP and other mechanistic model predictions of total PM_2.5_ concentrations in global cities are often substantially different from measured concentrations. Some studies (for example McDuffie et al. (2021)) calibrate their mechanistic model predictions to measurement and remote sensing data, but we do not do that here to provide a more accurate sense of the level of uncertainty surrounding our predictions.

**Fig. 2:**
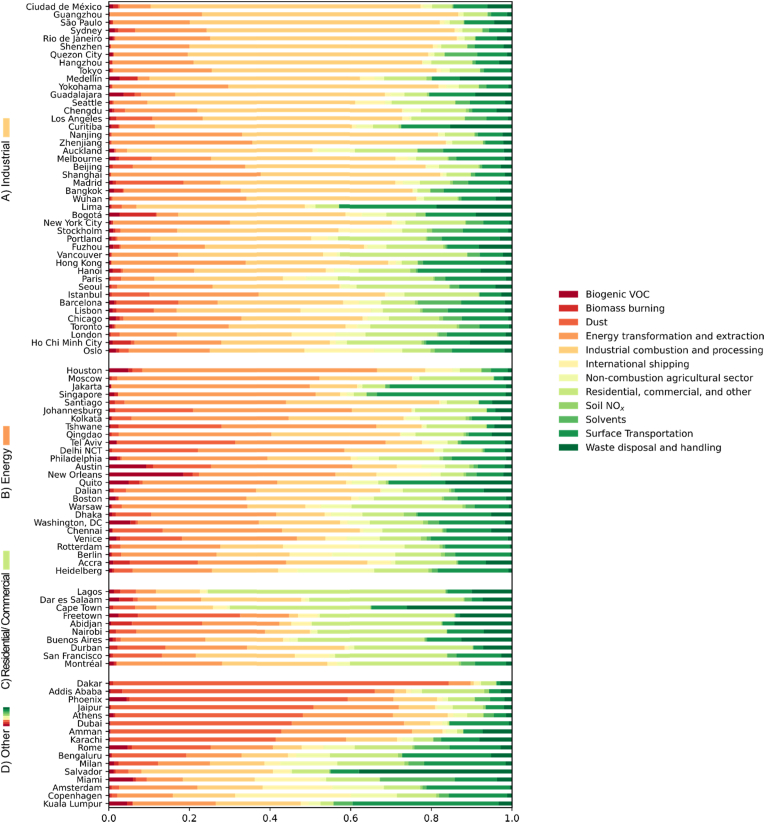
Proportions of total PM_2.5_ from 12 sources among 96 global cities, grouped by the largest sources: A) Industrial combustion and processing, B) Energy transformation and extraction, C) Residential, commercial, and other, and D) Other sectors.

The largest contributors to both total PM_2.5_ concentrations (Fig. 2) and PM_2.5_ concentrations caused by within-city emissions are most commonly industry, energy transformation and extraction, and residential and commercial activities. For example, 46% and 35% of cities have industry ranked the largest for total PM_2.5_ and PM_2.5_ concentrations caused by within-city emissions, respectively; 27% and 23% of cities have energy transformation and extraction ranked the largest for total PM_2.5_ and for PM_2.5_ concentrations caused by within-city emissions, respectively; and 10% and 24% of cities have residential and commercial activities ranked the largest for total PM_2.5_ and for PM_2.5_ concentrations caused by within-city emissions, respectively. The two largest sources of PM_2.5_ caused by out-of-city emissions are industry and energy. 43% of cities have industry ranked the largest source, and 30% of cities have energy ranked the largest source; however, the third largest source of PM_2.5_ caused by out-of-city emissions is dust, which is the largest out-of-city source in 15% of cities. Only 13% of cities have surface transportation ranked as the largest PM_2.5_ source caused by within-city emissions. Although industrial combustion/processing and energy transformation/extraction are the top PM_2.5_ sources in many cities, there is substantial variability in which emission sources contribute the most across the 96 cities. Thus, an important implication of these findings is that one-size-fits-all approaches to air quality management are unlikely to work across urban areas. Instead, management practice should consider local context including which sources dominate for that city.

### 3.2 Fraction of within-city emitted PM_2.5_ and city characteristics

As described in Section 2.4, we tested correlations between the fraction of PM_2.5_ caused by within-city emissions and city characteristics such as city size and urban GDP (Fig. S3). The results suggest low correlation between fraction of within-city emitted PM_2.5_ and city population, GDP and city area for total, primary and secondary PM_2.5_. However, there are positive correlations between the fraction of within-city emitted PM_2.5_ and population buffer fraction for total and secondary PM_2.5_.

There is no statistically significant difference in total PM_2.5_ concentration, city population, population buffer fraction or area among cities that have different top ranking emission sources (ANOVA p = 0.28, 0.71, 0.13, and 0.68, respectively). However, there is a statistical difference in the fraction of within-city generated PM_2.5_ among cities that have different top sources (p = 0.00), where cities with high fraction of within-city generated PM_2.5_ have top PM_2.5_ sources as residential and commercial, industrial, and energy, indicating these sources are likely generated locally. Additionally, there is a statistical difference in GDP among cities having different top sources (p = 0.045), consistent with the intuition that qualitatively different types of activities often occur in cities with different GDP levels. Along with the results shown in Fig. S3, our findings suggest if a city does not have other densely-populated areas nearby, it tends to have the most locally-generated PM_2.5_, whereas if a city has other densely populated areas nearby, it tends to have a smaller proportion of PM_2.5_ generated locally. The level of population buffer fraction is strongly associated with certain sources, as well as with total and secondary PM_2.5_ concentration. None of the urban parameters we investigated are well-correlated with the top PM_2.5_ source for a city. This finding suggests that atmospheric modeling holds value for understanding the local context of which sources contribute the most to local pollution.

### 3.3 Comparison of InMAP results with measurements and other studies

**Fig. 3:**
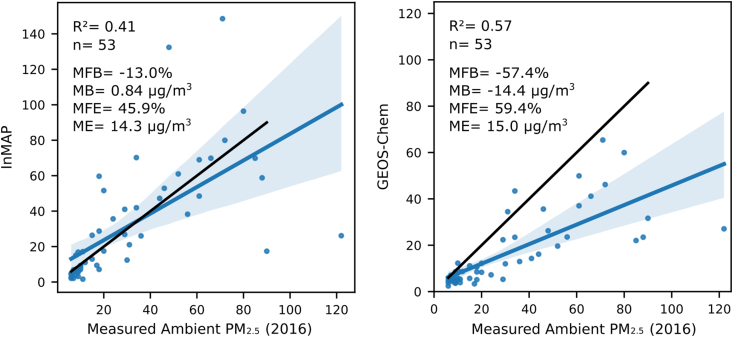
Comparison of InMAP predicted total population-weighted PM_2.5_ concentrations and measured total ambient PM_2.5_ concentrations (World Health Organization, 2016; left) and GEOS-Chem predicted total PM_2.5_ concentrations and measured total ambient PM_2.5_ concentrations (right) among 53 global cities. The blue line is a least-squares model fit and blue shaded areas indicate the 95% confidence interval of a least squares fit. The black line represents a 1:1 relationship. Error metric acronyms are defined in Table S3. (For interpretation of the references to colour in this figure legend, the reader is referred to the Web version of this article.)

We evaluate the InMAP predicted total population-weighted PM_2.5_ concentrations against measured total PM_2.5_ concentrations. There are 53 global cities that have both InMAP predictions and measured data collected by WHO (World Health Organization, 2016); results are shown in the first panel of Fig. 3. The r^2^ is 0.41 and bias and error are listed in Table S5. For the same cities, the model-measurement correlation is better for GEOS-Chem than for InMAP (r^2^ = 0.57 vs. 0.41 respectively; Fig. 3) but GEOS-Chem has larger error and bias (mean error: GEOS-Chem 15.0 µg/m^3^ vs. InMAP 14.3 µg/m^3^, and mean bias: GEOS-Chem −14.4 µg/m^3^ vs. InMAP 0.84 µg/m^3^) (Table S5). These results suggest that while GEOS-Chem provides a better mechanistic representation of the atmosphere overall, InMAP's higher resolution in urban areas helps it avoid underpredicting the increase in urban concentrations above the regional background.

**Fig. 4:**
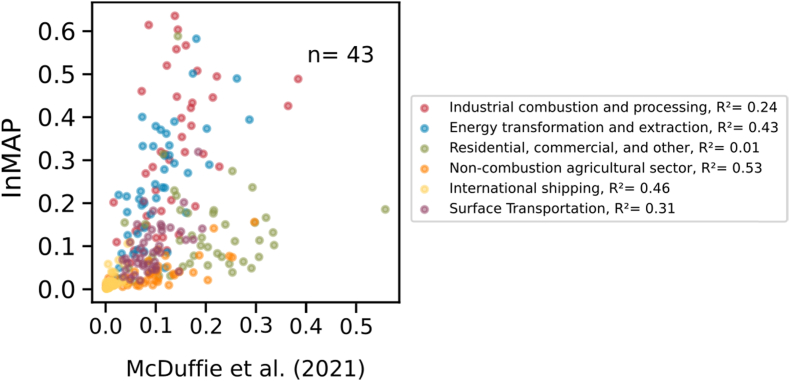
Comparison of fractions of total PM_2.5_ caused by different emission sources between InMAP (this study) and McDuffie et al. (2021) among 43 global cities.

We additionally compare the InMAP predicted total PM_2.5_ concentrations using satellite derived PM_2.5_ concentrations (Southerland et al., 2021) among 91 cities in Fig. S4 (r^2^ = 0.15), as well as using modeled total PM_2.5_ concentrations reported by McDuffie et al. (2021) in the first panel of Fig. S5 (r^2^ = 0.23). (Note that total PM_2.5_ concentrations reported by McDuffie et al. (2021) are calibrated to measurement and remote sensing data, which explains their good agreement with measurement data.) We compare our estimates for fractions of total PM_2.5_ caused by six common emission sources with those reported by McDuffie et al. (2021) in Fig. 4. Most fractions of these emission sources show relatively good agreement (r^2^: 0.24–0.53) between the two studies except the fraction of PM_2.5_ from residential, commercial and other (r^2^ = 0.01). We also compare InMAP fractions of total PM_2.5_ concentrations caused by within-city emissions with these fractions from 17 European cities (Thunis et al., 2021) as shown in Fig. S6 (r^2^ = 0.18). Additionally, we compare the fractions of total PM_2.5_ caused by eight common emission sources, as well as the fractions of total PM_2.5_ concentrations caused by these eight emission sources generated by within-city emissions to that study in Figs. S7 and S8. There are no correlations between InMAP predictions and predictions by Thunis et al. (2021), except the fractions of total PM_2.5_ caused by agricultural and residential sources (the differences in emission source categories are listed in Table S6). We also evaluate the InMAP within-city fractions for total PM_2.5_ against zero-out simulations with GEOS-Chem conducted in five cities and find good agreement (r^2^ = 0.67) when InMAP is run at the coarser resolution used by GEOS-Chem (Fig. S9). Model comparison is described in more detail in the supporting information.

## 4 Discussion

In this study, we have produced estimates of PM_2.5_ concentration, its source composition, and contribution of in-city vs. out-of-city sources—the latter of which was not previously available in many global cities. This information can be useful as a screening tool and in many cases may be robust enough to inform policy action to enact more effective strategies for improving public health. We find that although industrial- and energy-related sources are the largest contributors to PM_2.5_ in a plurality of cities, there is considerable diversity among cities in which source types are most important (Fig. 2). Because there is considerable uncertainty inherent in the results presented here, in some cases it may be advisable to leverage these results to obtain more precise estimates using a more mechanistically-detailed air quality model and locally-produced emission inventories before taking policy action, to the extent that resources are available to support the additional analysis.

Beyond the specific results for individual cities presented in the supporting information and summarized above, we would like to call the reader's attention to several salient points that emerge from this analysis.

The first point is that we did not find any strong patterns among cities that could predict which emission sources contribute most to a city's PM_2.5_ concentrations, or how much of a city's ambient PM_2.5_ concentration originates from emissions within the city boundary (Fig. S3). To some extent, this contradicts previous findings by Apte et al. (2012); this discrepancy may be explained by the more detailed model of the atmosphere used here. The implication of this point is that it is necessary to perform atmospheric modeling in a given city to get a realistic estimate of the sources of that city's pollution—there do not seem to be any shortcuts. Since it may not be practical to carry out urban-level air quality simulations in a large number of cities using a comprehensive model like GEOS-Chem, this underscores the utility of reduced-complexity models such as InMAP.

The second point is that urban air quality analyses require urban emissions inventories. As described above, we use spatial surrogates—mainly based on OpenStreetMap data—to allocate 0.5 × 0.5° CEDS emissions to much smaller InMAP grid cells. This spatial downscaling is important: in an analysis comparing InMAP and GEOS-Chem predicted contributions of within-city emissions for a subset of five of the 96 cities studied here (i.e. Johannesburg, Buenos Aires, Addis Ababa, Chengdu, and Guadalajara), we found that the r^2^ value between GEOS-Chem and InMAP predictions when InMAP used emissions at their native resolution was 0.67, but when InMAP used the same emissions downscaled with the spatial surrogates described above, the r^2^ value decreased to 0.2 (Fig. S9, methods in supporting text). This implies that the use of high-resolution emissions provides information that couldn't be reproduced by—for example—applying a correction factor to simulation results based on low-resolution emissions.

Building on the second point, the third point is that in this analysis the emission inventory appears to be a larger source of potential error than the choice of air quality model. For example, the r^2^ value for total concentration predictions in five cities between GEOS-Chem and InMAP when using the same emission inventory is 0.98 (Fig. S9), but the r^2^ value between GEOS-Chem and InMAP for the fractional contribution of Residential, Commercial, and Other emissions in 43 cities when using different versions of the CEDS inventory (Hoesly et al., 2018 vs. McDuffie et al., 2021) at different spatial resolutions is 0.01 (Fig. 4).

As described above, high-resolution emissions estimates are important for urban-scale analysis. The fourth point is that downscaling existing global inventories using spatial surrogates can only yield improvements up to a certain point. The global CEDS inventory used here is mainly based on national emissions estimates that are themselves downscaled to a 0.5 × 0.5° grid using mainly population density estimates. This can lead to spatial misallocations that cannot be fixed by further downscaling. For example, using the national-population-based spatial allocation method above in a country with substantial residential coal emissions could allocate a plurality of those emissions to the cosmopolitan capital city, where in reality there are relatively few residential coal emissions owing to the capital's relatively high level of affluence. The next generation of global emissions inventories may benefit from the emergence of new streams of local data—for example from smart phones and satellites—in combination with local expertise facilitated by networks of cities like C40.

## CRediT authorship contribution statement

**Mei W. Tessum:** Formal analysis, Investigation, Resources, Writing – original draft, Writing – review & editing, Visualization. **Susan C. Anenberg:** Validation, Writing – review & editing, Funding acquisition, Project administration. **Zoe A. Chafe:** Conceptualization, Validation, Writing – review & editing, Funding acquisition. **Daven K. Henze:** Validation, Writing – review & editing. **Gary Kleiman:** Conceptualization, Writing – review & editing. **Iyad Kheirbek:** Conceptualization, Validation, Writing – review & editing, Funding acquisition, Project administration. **Julian D. Marshall:** Conceptualization, Resources, Writing – review & editing, Funding acquisition. **Christopher W. Tessum:** Conceptualization, Methodology, Software, Validation, Investigation, Resources, Data curation, Writing – review & editing.

## Declaration of competing interest

The authors declare the following financial interests/personal relationships which may be considered as potential competing interests:
