# Supplementary material for: Sources of ambient PM2.5 exposure in 96 global cities
Source: Atmos Environ (1994). 2022 Oct 1;286:119234. doi: 10.1016/j.atmosenv.2022.119234 (PMC9297293; doi:10.1016/j.atmosenv.2022.119234)
Supplement: Multimedia component 1 [file mmc1.docx]

**Supplementary material**

**Title**

Sources of ambient PM2.5 exposure in 96 global cities

**Authors**

# Mei W. Tessum^a^, Susan C. Anenberg^b^, Zoe Chafe^c^, Daven K. Henze^d^, Gary Kleiman^e^, Iyad Kheirbek^c^, Julian D. Marshall^f^, Christopher W. Tessum^g,*^

# ^a^Department of Agricultural and Biological Engineering, University of Illinois at Urbana-Champaign, Urbana, Illinois, United States

# ^b^Department of Environmental and Occupational Health, George Washington University, Washington, DC, United States

# ^c^C40 Cities Climate Leadership Group Inc., New York, New York, United States

^d^Department of Mechanical Engineering, University of Colorado, Boulder, Colorado, United States

# ^e^Orbis Air, LLC. Concord, Massachusetts, United States

# ^f^Department of Civil and Environmental Engineering, University of Washington, Seattle, Washington, United States

# ^g^Department of Civil and Environmental Engineering, University of Illinois at Urbana-Champaign, Urbana, Illinois, United States

# * Email and address for correspondence:

# Christopher W. Tessum

# ctessum@illinois.edu

# 3213 Newmark Civil Engineering Building,

205 North Mathews Avenue Urbana, IL 61801, USA

**Contents**

- Spatial surrogates evaluation
- InMAP Model prediction comparison with other studies with emission source information
- Dataset S1. City source apportionment data
- Table S1. Pollutant labels in CEDs vs. InMap
- Table S2. List of OSM definition for different roadway types
- Table S3. Definitions of error and bias metrics used to evaluate InMAP model prediction
- Table S4. Summary among the 96 cities of InMAP predicted ambient primary and secondary PM_2.5_ concentration from all and within-city sources
- Table S5. Model prediction comparison with measured PM_2.5_ concentration
- Table S6. Differences in emission source categories
- Figure S1. The input data, output data, and modeling tools used in this study.
- Figure S2. The InMAP grid used for global simulations
- Figure S3. Relationships between the fraction of PM_2.5_ originating from within city emission sources and different city characteristics
- Figure S4. Comparison of InMAP predicted total PM_2.5_ concentrations and GBD PM_2.5_ concentrations among 91 global cities.
- Figure S5. Comparisons of InMAP predicted total PM_2.5_ concentrations and McDuffie *et al.* (2021) concentrations among 43 global cities (left) and McDuffie *et al.* (2021) concentrations and measured total ambient PM_2.5_ concentrations (WHO 2016, right) among 51 cities.
- Figure S6. Comparison of fractions of total PM_2.5_ caused by within-city emission sources between InMAP and EC PM_2.5_ atlas data among 17 European cities (Amsterdam, Athens, Barcelona, Berlin, Copenhagen, Heidelberg, Lisbon, London, Madrid, Milan, Oslo, Paris, Rome, Rotterdam, Stockholm, Venice and Warsaw).
- Figure S7. Comparison of fractions of total PM_2.5_ concentrations caused by 8 emission sources between InMAP and EC PM_2.5_ atlas data among 17 European cities (Amsterdam, Athens, Barcelona, Berlin, Copenhagen, Heidelberg, Lisbon, London, Madrid, Milan, Oslo, Paris, Rome, Rotterdam, Stockholm, Venice and Warsaw).
- Figure S8. Comparison of fractions of within-city PM_2.5_ concentrations caused by 8 emission sources between InMAP and EC PM_2.5_ atlas data among 17 European cities (Amsterdam, Athens, Barcelona, Berlin, Copenhagen, Heidelberg, Lisbon, London, Madrid, Milan, Oslo, Paris, Rome, Rotterdam, Stockholm, Venice and Warsaw).
- Figure S9. Comparison of A) total PM_2.5_ concentration, B) fractions of within-city PM_2.5_ concentrations with low resolution InMAP spatial surrogates, and C) fractions of within-city PM_2.5_ concentrations with high resolution InMAP spatial surrogates between InMAP and GEOS-Chem model among 5 global cities (Johannesburg, Buenos Aires, AddisAbaba, Chengdu, and Guadalajara).

**Spatial surrogates evaluation**

We compared estimates of total PM_2.5_ and within-city fractions from InMAP and with those generated from GEOS-Chem (Figure S7) for five cities (Johannesburg, Buenos Aires, AddisAbaba, Chengdu, and Guadalajara) . For this comparison, GEOS-Chem was run at the global 2°x2.5° resolution and within-city fractions were simply estimated through perturbation simulations in which the anthropogenic emissions in the individual grid cell containing a city-center were set to zero. Both models used emission estimates from the ECLIPSE inventory for 2016 (Stohl *et al.*, 2015). InMAP within-city fractions were estimated two ways -- first using spatial surrogates for the emissions that were degraded to the same spatial resolution of that as GEOS-Chem (low-res), and again using high resolution spatial surrogates at the 3 x 4 km scale (high-res). The r^2^ is 0.98 for predicting total PM_2.5_ concentrations, which is to be expected given the use of GEOS-Chem (albeit a different version) as a basis for InMAP. The r^2^ is 0.67 for predicting fraction of within-city PM_2.5_ when InMAP using low resolution spatial surrogates; this confirms that InMAP is able to reproduce the source attribution one would expect at the coarse scale of the global GEOS-Chem model.

When using the InMAP high resolution spatial surrogates, the r^2^ drops to 0.20, which shows that the higher resolution estimates provide unique information on spatial variability.

**InMAP Model prediction comparison with other studies that include emission source information**

We found a lack of correlation (r^2^=0.18, Figure S5) among 17 European cities, which is somewhat expected as there are several differences in methods between a European Commission study (Thunis *et al*. 2017) and the current study. First, the two studies do not use identical source categories; the main differences in source categories are listed in Table S6. Therefore we only see correlations between fractions of total PM_2.5_ caused by transportation (r^2^=0.56), agriculture (r^2^=0.48) and energy sector (r^2^=0.33) (Figure S6). Secondly, the two studies use different emissions inventories (CAMS V4.2 in the EC study and CEDS in this study) for different years (2015 in the EC and 2014 in this study), as well as different air quality models (SHERPA in the EC study and InMAP in this study). Additionally, the temporal domain and spatial resolution are different between EC PM_2.5_ Atlas and this study. The spatial resolution adopted in the EC PM_2.5_ Atlas is that of SHERPA (approximately 10×10 km) which is a coarser grid for some of the urban areas, while the InMAP model adopts a finer grid (about 3×4 km at the equator) at populated urban areas. Furthermore, this study calculates population-weighted concentrations within each city, whereas the EC study performs source apportionment at a single location in the center of the city. The differences of temporal domain and spatial resolution, city boundary and population weighing on concentration may explain why there are only correlations seen between the fractions of total PM_2.5_ concentrations caused by residential, commercial, and other (r^2^=0.32) and shipping (r^2^=0.38) sectors that generated by within-city emissions (Figure S7). The EC study uses an emission inventory specific to Europe, which would generally be expected to be more accurate than the global emissions inventory in this study, but the current study has other methodological advantages that makes it unclear which study could be expected to provide more reliable estimates. Additionally, we found some correlations between McDuffie *et al.* (2021) and the current study among 43 global cities. There are stronger correlations between the two models among PM_2.5_ concentrations caused by international shipping, agriculture, surface transportation, and energy industry compared to other sources. Although some of the differences in prediction are owing to different emission inventory and spatial resolution, it suggests that InMAP provides more source contributions as well as more detailed estimates of PM_2.5_ source contributions in certain sources than certain models (i.e., the EC study), for example, sources of international shipping, industry, surface transportation, and energy transformation and extraction.

**Dataset S1: City source apportionment data**

This file contains population-weighted PM_2.5_ concentrations caused by different source types and emitted pollutants.

All values are ug m^-3^ of PM_2.5_; the different pollutant names refer to the pollutant that was emitted and eventually turned into PM_2.5_.

Columns:

- city: The name of the city
- source: Emission source type
- emission: The type of pollutant emitted
- Total: Total PM_2.5_ concentration caused by emissions both within and outside the city
- Within City: PM_2.5_ concentration caused by emissions within the city
- Outside of City: PM_2.5_ concentration caused by emissions outside of the city.

Table S1. Pollutant label in CEDs vs. InMap

| **CEDs** | **InMap** |
| --- | --- |
| SO2 | SOx |
| NOx | NOx |
| NMVOC components* | VOC |
| BC and OC | PM2.5 |
| NH3 | NH3 |

* includes ‘ALD2’, ‘ALK4_butanes’, ‘ALK4_hexanes’, ‘ALK4_pentanes’, ‘BENZ’, ‘BUTENE’, ‘C2H2’, ‘C2H4’, ‘C2H6’, ‘C3H8’, ‘CH2O’, ‘CHC’, ‘EOH’, ‘ESTERS’, ‘ETHERS’, ‘MEK’, ‘OTHER_AROM’, ‘OTHER_VOC’, ‘PRPE’, ‘TMB’, ‘TOLU’, ‘XYLE’, and ‘HCOOH’ (which are considered secondary organic aerosol (SOA) precursors by GEOS-Chem)

Table S2. List of OSM definition for different roadway types

| **Roadway Types** | **OSM Definition** |
| --- | --- |
| Residential roadways | Roads which serve as an access to housing, without function of connecting settlements. Often lined with housing. |
| Motorways | A restricted access major divided highway, normally with 2 or more running lanes plus emergency hard shoulder. Equivalent to the Freeway, Autobahn, etc. |
| Trunk roadways | The most important roads in a country’s system that are not motorways. (Need not necessarily be a divided highway.) |
| Primary roadways | The next most important roads in a country’s system. (Often link larger towns.) |
| Secondary roadways | The next most important roads in a country’s system. (Often link towns.) |
| Tertiary roadways | The next most important roads in a country’s system. (Often link smaller towns and villages). |
| Service roads | For access roads to, or within an industrial estate, camp site, business park, car park etc. |
| Unclassified roads | The least important through roads in a country’s system – i.e. minor roads of a lower classification than tertiary, but which serve a purpose other than access to properties. |

Table S3. Definitions of errors and bias used to evaluate InMAP model prediction

| **Metrics** | **Equation** | **Note** |
| --- | --- | --- |
| Mean fractional bias  (MFB) | 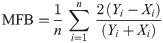 | *i* corresponds to one of *n* comparisons, and *X* and *Y* are the annual average modeled or measured values we are comparing |
| Mean bias  (MB) | 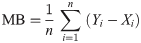 |  |
| Mean fractional error  (MFE) | 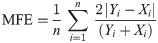 |  |
| Mean error  (ME) | 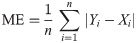 |  |

Table S4. Summary among the 96 cities of InMAP predicted ambient primary and secondary PM_2.5_ concentration from all and within-city sources

|  | **fraction of PM_2.5_ attributable to within-city emissions** | | | **PM_2.5_ concentration (**μg/m^3^**), all sources** | | | **PM_2.5_ concentration (**μg/m^3^**), within-urban sources only** | | |
| --- | --- | --- | --- | --- | --- | --- | --- | --- | --- |
| **Measure** | **for total PM_2.5_** | **for primary PM_2.5_** | **for secondary PM_2.5_** | **total** | **primary** | **secondary** | **total** | **primary** | **secondary** |
| Mean | 0.37 | 0.44 | 0.37 | 29.2 | 7.0 | 22.2 | 11.9 | 2.6 | 9.4 |
| Standard Deviation | 0.22 | 0.23 | 0.22 | 29.9 | 10.1 | 25.9 | 16.1 | 2.5 | 14.5 |
| Minimum | 0.04 | 0.05 | 0.03 | 1.62 | 0.19 | 1.4 | 0.17 | 0.05 | 0.12 |
| 25th Percentile | 0.21 | 0.25 | 0.20 | 7.57 | 1.46 | 6.0 | 1.9 | 0.40 | 1.1 |
| 50th Percentile | 0.33 | 0.44 | 0.32 | 17.4 | 4.0 | 13.1 | 7.3 | 1.8 | 3.6 |
| 75th Percentile | 0.52 | 0.62 | 0.53 | 40.1 | 9.8 | 27.8 | 14.2 | 4.0 | 10.3 |
| Maximum | 0.95 | 0.97 | 0.95 | 148.6 | 83.5 | 131.6 | 81.5 | 10.4 | 77.4 |

Table S5. Model prediction comparison with measured PM_2.5_ concentration

| Comparison | Mean fractional bias  (MFB) | Mean bias  (MB) | Mean fractional error  (MFE) | Mean error  (ME) |
| --- | --- | --- | --- | --- |
| InMAP vs. measured PM_2.5_ conc. | -13.0% | 0.84 | 45.9% | 14.3 |
| GEOS-Chem vs. measured PM_2.5_ conc. | -57.4% | -14.4 | 59.4% | 15.0 |

Table S6. Differences in emission source categories

| **Source** | **EC PM_2.5_ Atlas** | **InMAP** |
| --- | --- | --- |
| **Energy** | Extraction and distribution of fossil fuels | Electricity production, heat production, other energy transformation, related fugitive emissions, and fossil fuel fires |
| **Industry** | Industrial combustion and industrial processes | Industrial combustion and processes, NOT including energy transformation and extraction |
| **Residential** | Emissions from combustion in domestic heating appliances (open fireplaces, stoves, etc.), medium and single-house boilers, cooking and heating stoves in commercial, institutional and residential activities | All residential, commercial, and other |
| **Solvent** | Solvent use | Solvent used in degreasing and cleaning |
| **Transportation** | Exhaust and evaporative emissions from light and heavy-duty vehicles and motorcycles, non-exhaust PM emissions due to road abrasion of tires and brake wear, as well as from the off-road emissions sector (e.g., agricultural and construction equipment). Aviation. | All surface transportation, including road, rail and other |
| **Shipping** | Sailing boats, motorboats, inland goods carrying vessels, international inland gateways as well as national and international sea traffic. | VOCs from oil tanker loading/leakage |
| **Waste** | Waste treatment and disposal | Solid waste disposal, waste combustion, wastewater handling, and other |
| **Agriculture** | Emissions from livestock, fertilizer use and field burning of agricultural residuals | Non-combustion agricultural sector |


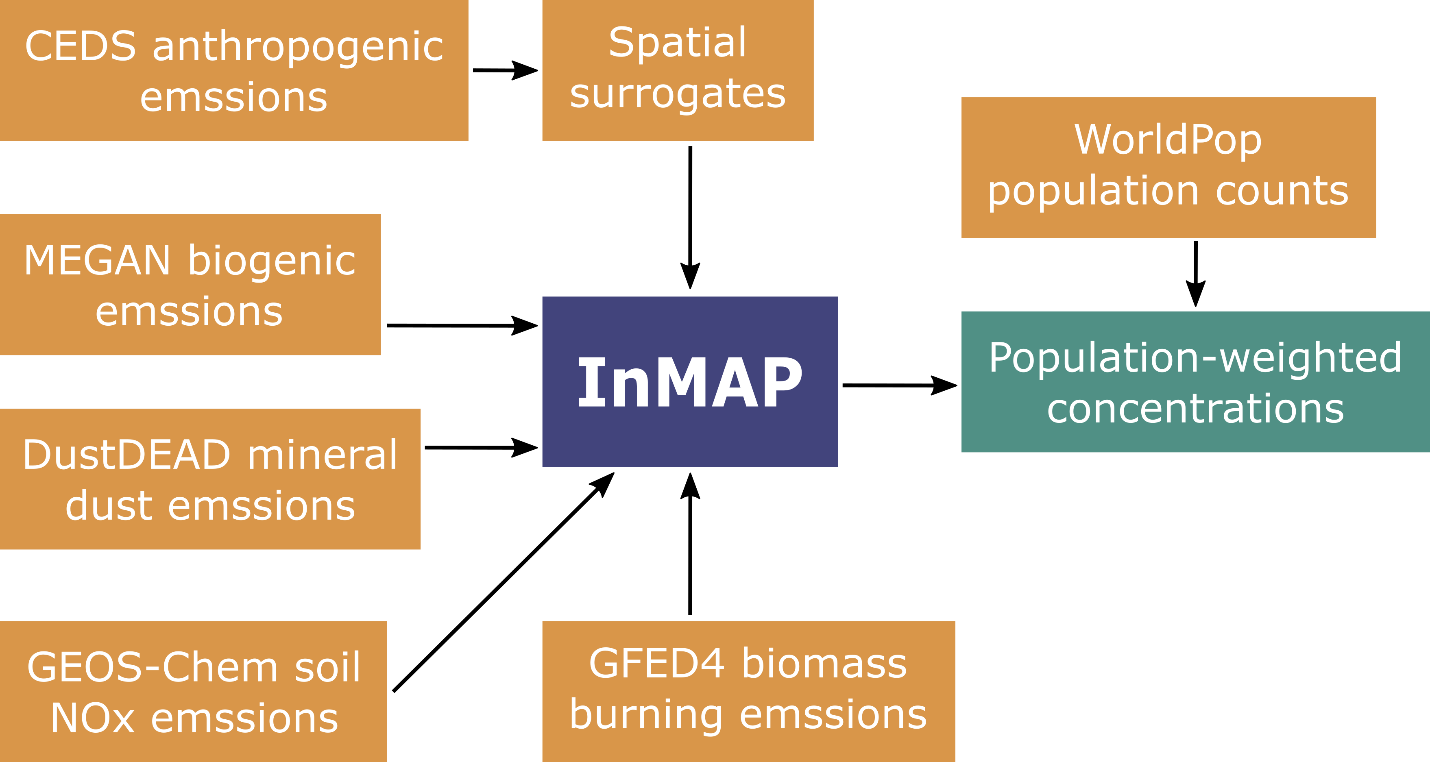


Figure S1. The input data, output data, and modeling tools used in this study.


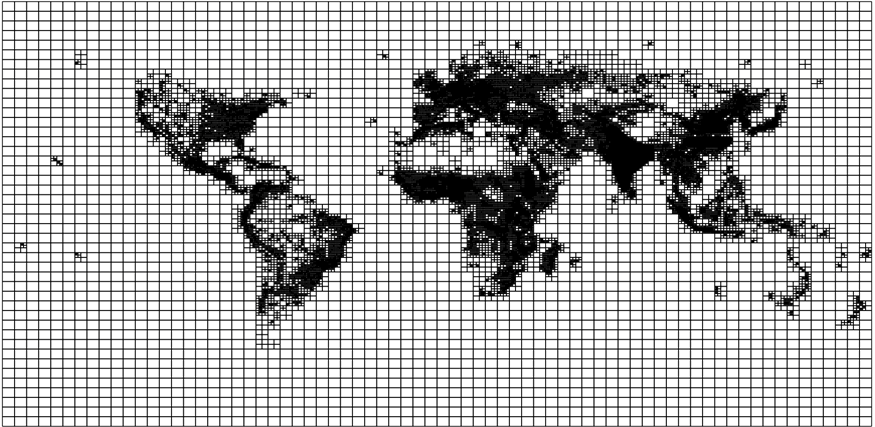


Figure S2. The InMAP grid used for global simulations.


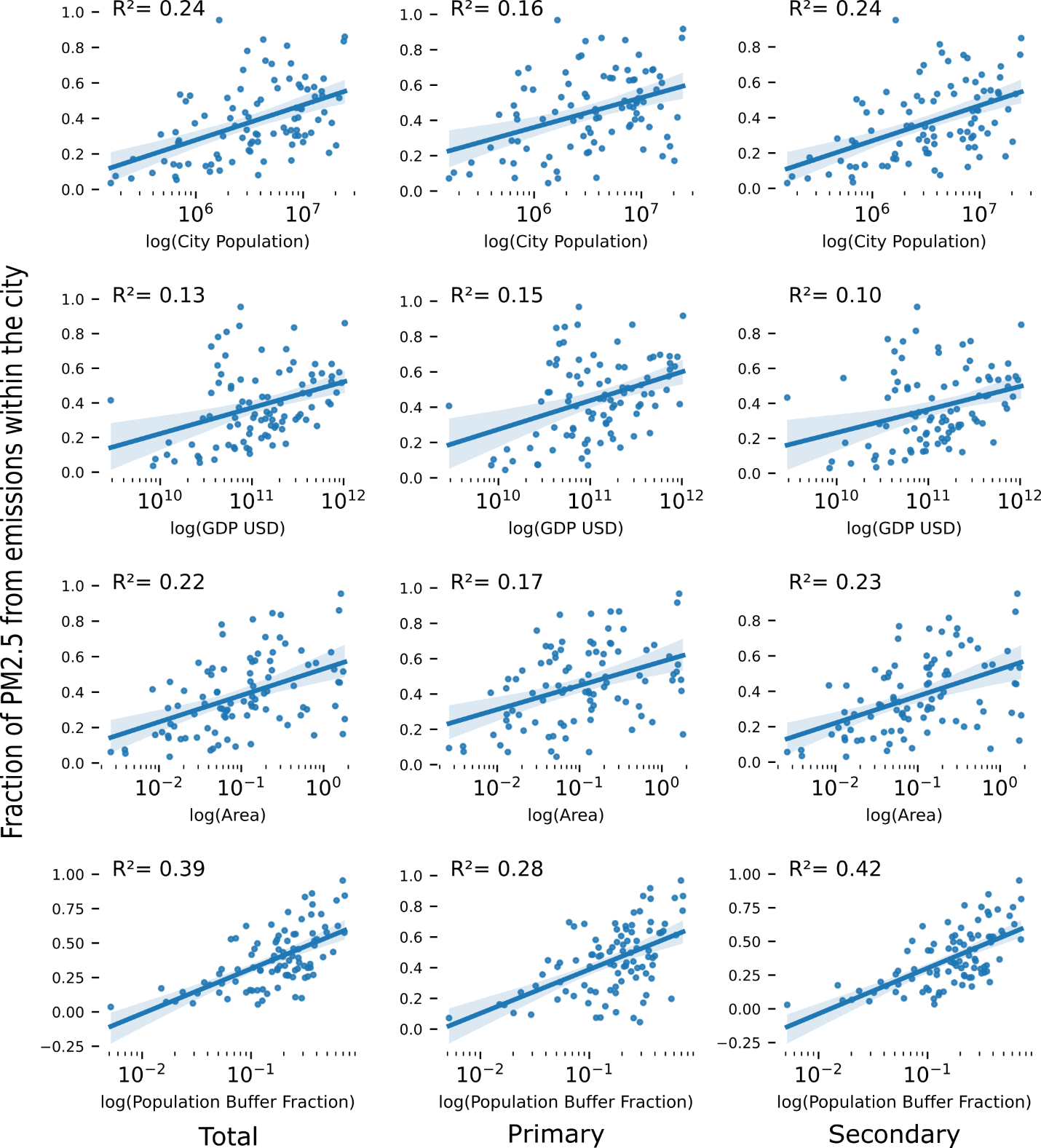


Figure S3. Relationships between the fraction of PM_2.5_ originating from within city emission sources and different city characteristics: city population (log scale), GDP (in USD, log scale), city area (in degree^2^, log scale), and population buffer fraction* (log scale) for total PM_2.5_, primary PM_2.5_ and secondary PM_2.5_ among 96 global cities. The blue line is a least-squares model fit and blue shaded areas indicate the 95% confidence interval of least squares fit. The black line represents a 1:1 relationship.

* Population buffer fraction is the fraction of city population divided by total population within a 200 km radius of the city center.


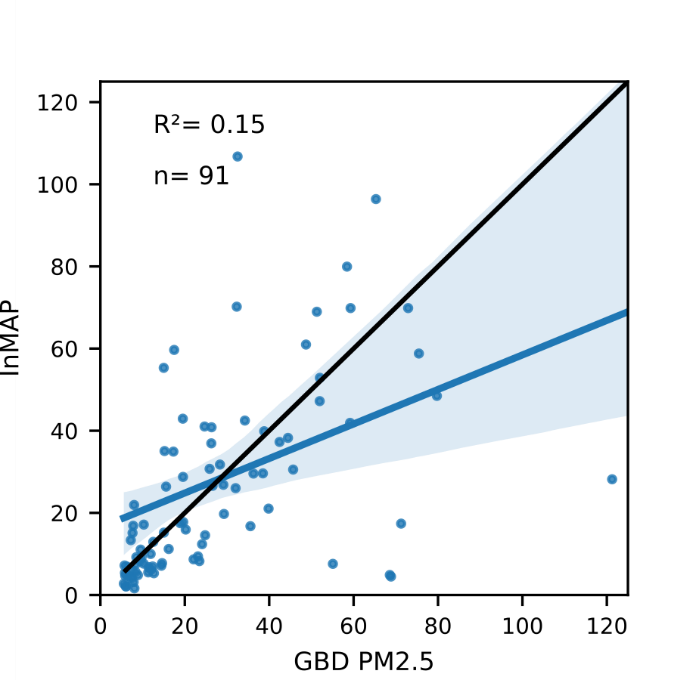


Figure S4. Comparison of InMAP predicted total PM_2.5_ concentrations and Southerland *et al*. (in prep) PM_2.5_ concentrations among 91 global cities. The blue line indicates the expected value of a least-squares fit and blue shaded areas indicate 95% confidence interval of least squares fit. The black line is the 1:1 line.


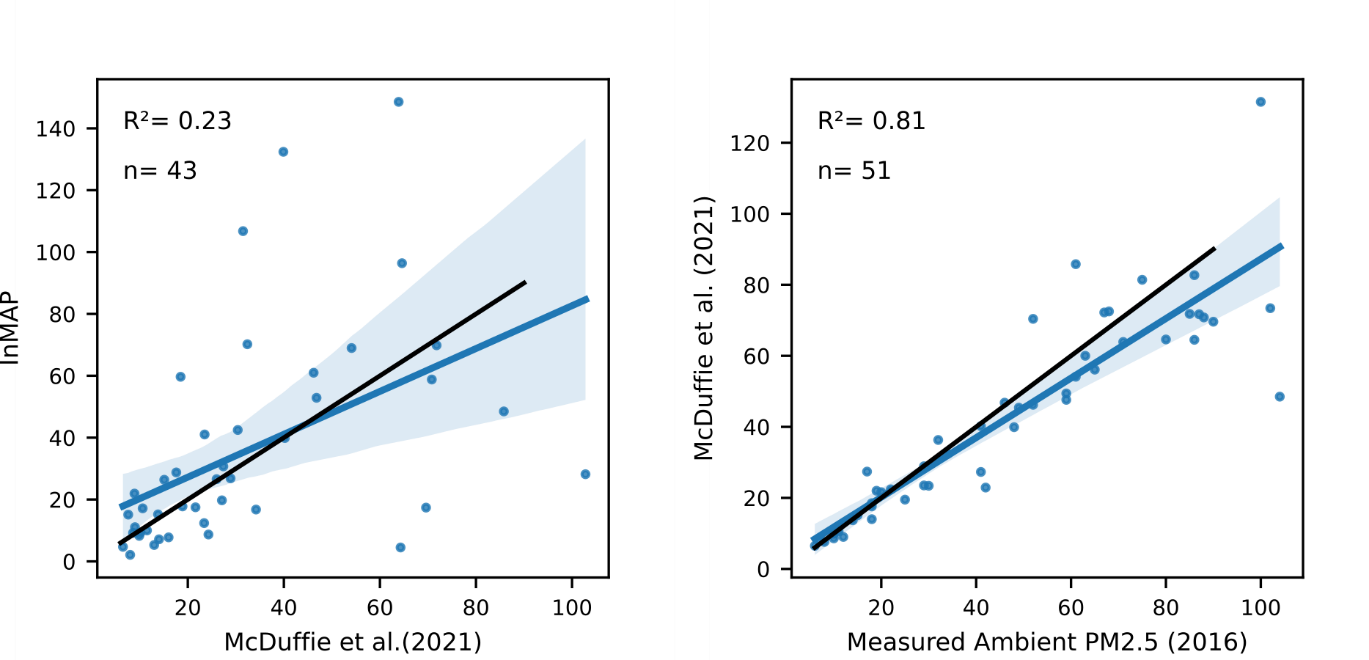


Figure S5. Comparisons of InMAP predicted total PM_2.5_ concentrations and McDuffie *et al.* (2021) concentrations among 43 global cities (left) and McDuffie *et al.* (2021) concentrations and measured total ambient PM_2.5_ concentrations (WHO 2016, right) among 51 cities. The blue line indicates the expected value of a least-squares fit and blue shaded areas indicate 95% confidence interval of least squares fit. The black line is the 1:1 line. Note that total PM_2.5_ concentrations reported by McDuffie *et al.* (2021) are calibrated to measurement and remote sensing data, but InMAP concentrations are not.


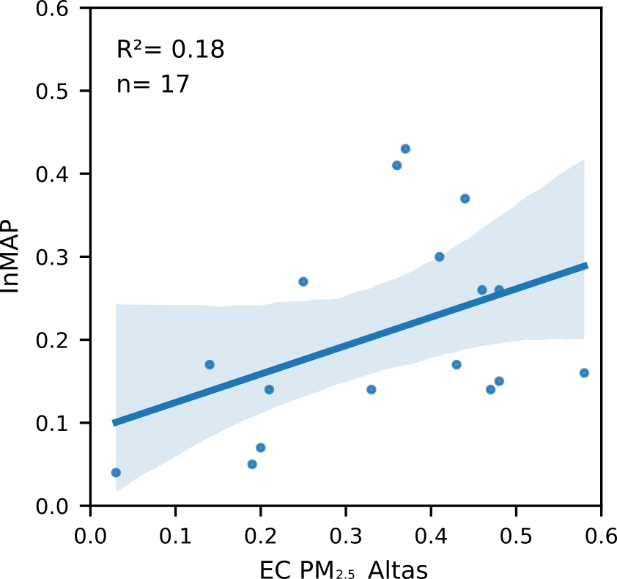


Figure S6. Comparison of fractions of total PM_2.5_ caused by within-city emission sources between InMAP and EC PM_2.5_ atlas data among 17 European cities (Amsterdam, Athens, Barcelona, Berlin, Copenhagen, Heidelberg, Lisbon, London, Madrid, Milan, Oslo, Paris, Rome, Rotterdam, Stockholm, Venice and Warsaw). The blue line indicates the expected value of a least-squares fit and blue shaded areas indicate 95% confidence interval of least squares fit.


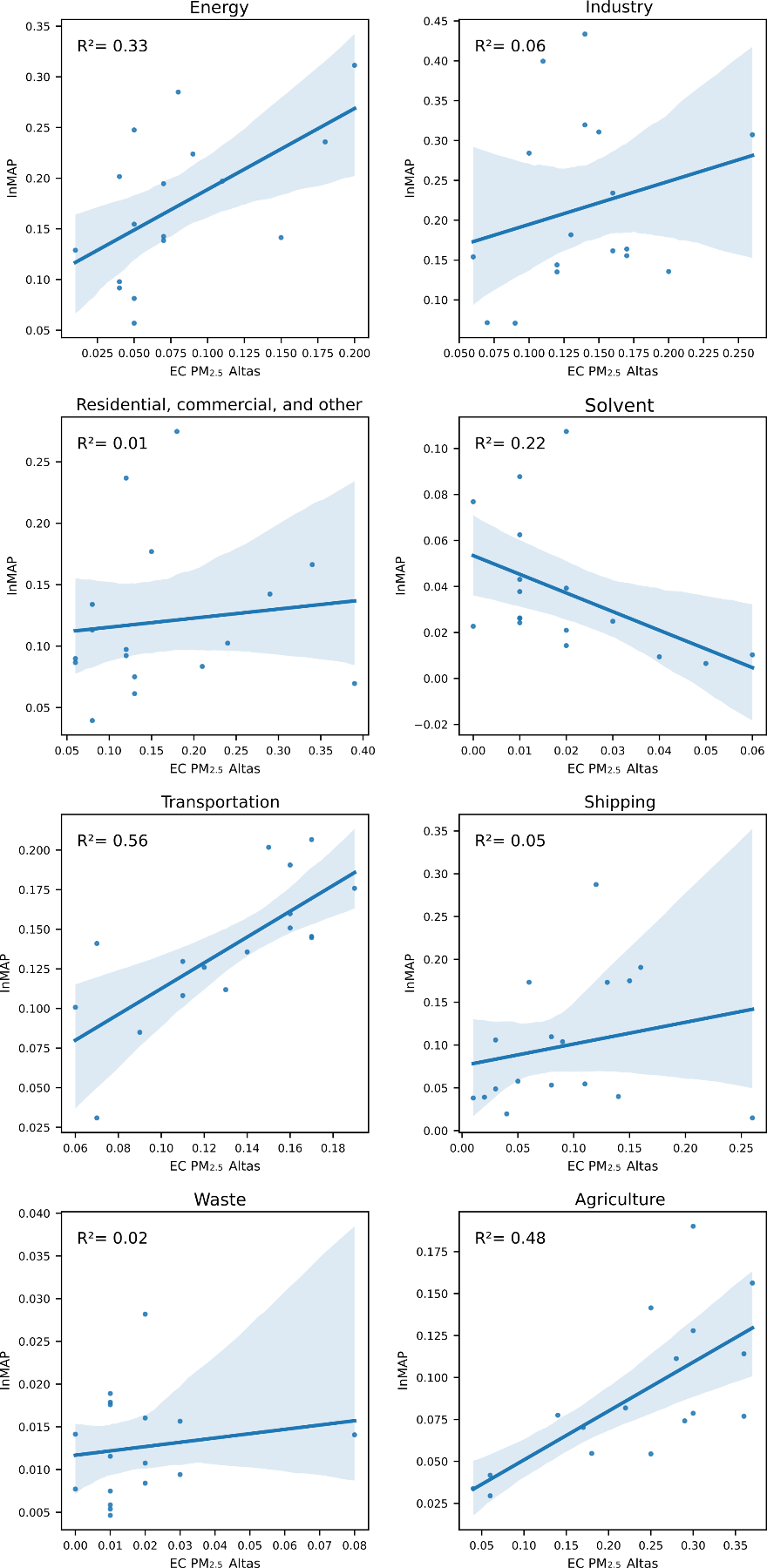


Figure S7. Comparison of fractions of total PM_2.5_ concentrations caused by 8 emission sources between InMAP and EC PM_2.5_ atlas data among 17 European cities (Amsterdam, Athens, Barcelona, Berlin, Copenhagen, Heidelberg, Lisbon, London, Madrid, Milan, Oslo, Paris, Rome, Rotterdam, Stockholm, Venice and Warsaw)


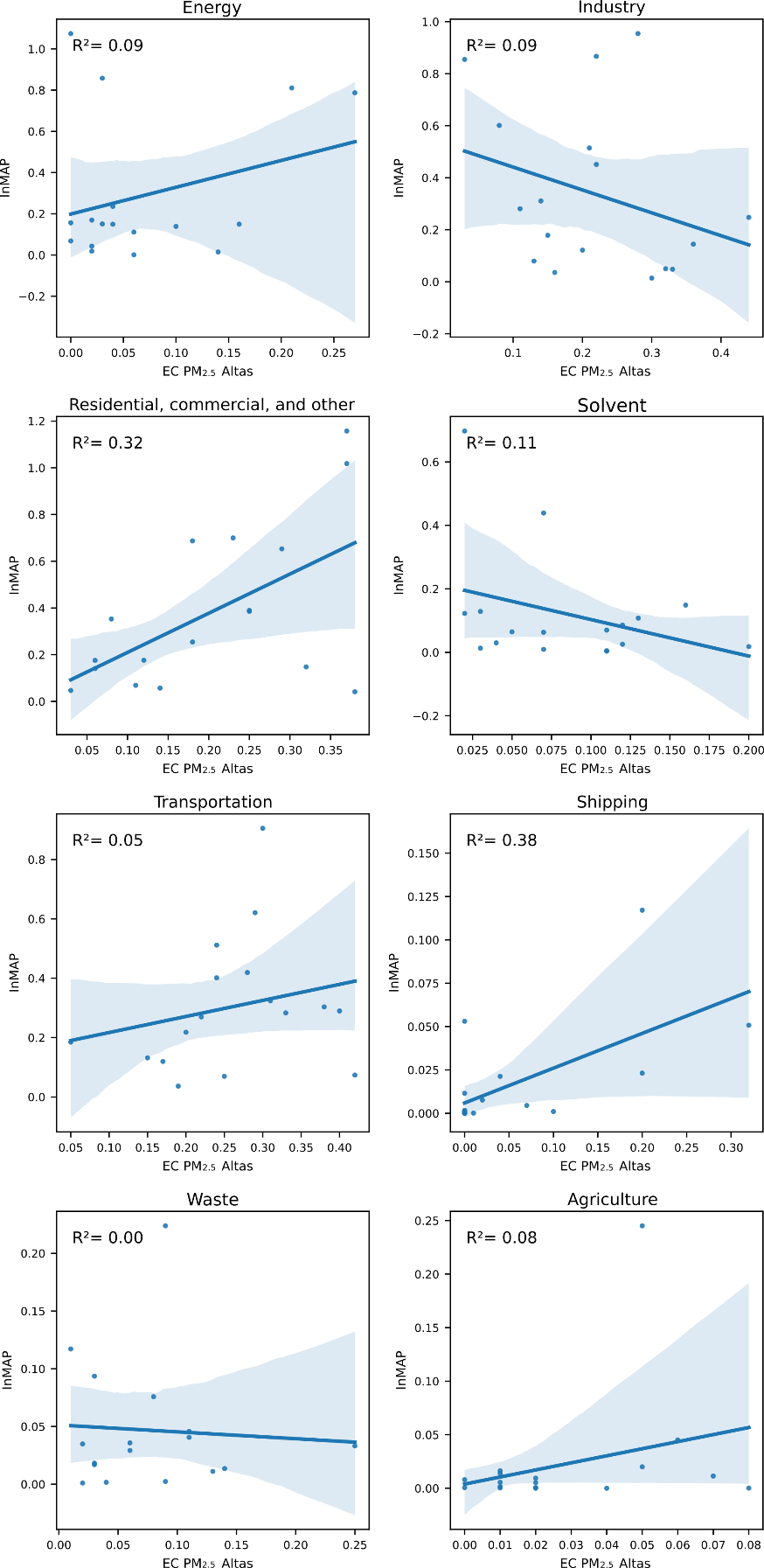


Figure S8. Comparison of fractions of within-city PM_2.5_ concentrations caused by 8 emission sources between InMAP and EC PM_2.5_ atlas data among 17 European cities (Amsterdam, Athens, Barcelona, Berlin, Copenhagen, Heidelberg, Lisbon, London, Madrid, Milan, Oslo, Paris, Rome, Rotterdam, Stockholm, Venice and Warsaw)


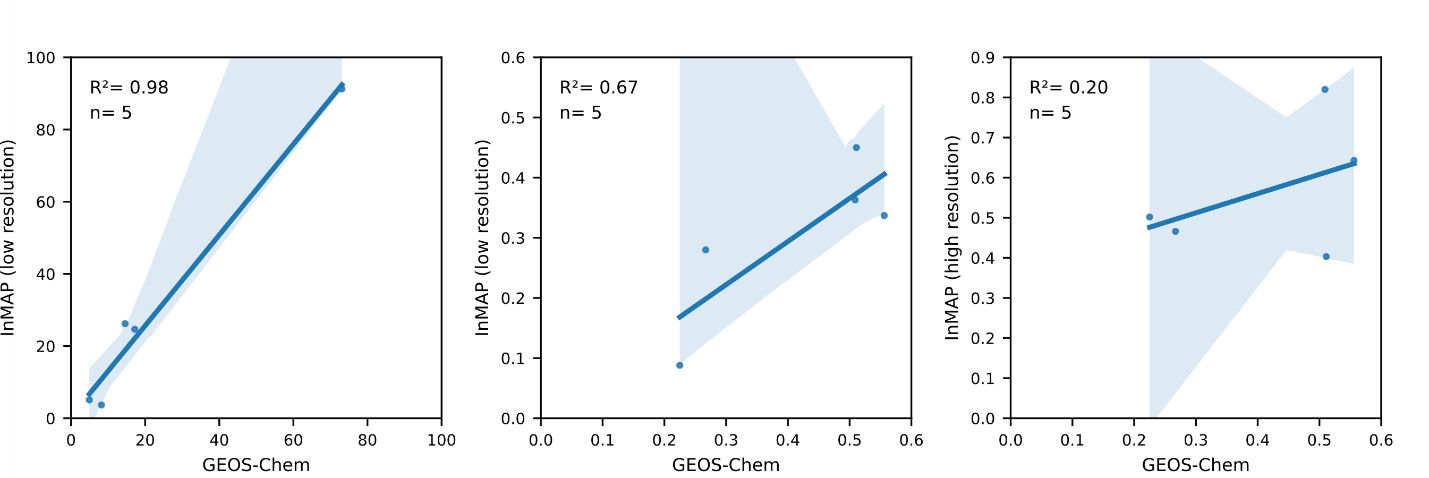


Figure S9. Comparison of A) total PM_2.5_ concentration, B) fractions of within-city PM_2.5_ concentrations with low resolution InMAP spatial surrogates, and C) fractions of within-city PM_2.5_ concentrations with high resolution InMAP spatial surrogates between InMAP and GEOS-Chem model among 5 cities (Johannesburg, Buenos Aires, AddisAbaba, Chengdu, and Guadalajara). The blue line indicates the expected value of a least-squares fit and blue shaded areas indicate 95% confidence interval of least squares fit.
